# SECONDARY USE OF NIH AD/ADRD DATA RESOURCES FOR BIOMEDICAL RESEARCH

**DOI:** 10.1093/geroni/igad104.3583

**Published:** 2023-12-21

**Authors:** Sharna Tingle, Damali Martin, Jennie Larkin

**Affiliations:** National Institute on Aging, Bethesda, Maryland, United States; National Institute on Aging, Bethesda, Maryland, United States; National Institute on Aging, Bethesda, Maryland, United States

## Abstract

People are living longer than ever before, and the world’s population aged 60 and older is expected to increase from 1 billion to 2.1 billion by 2050. As populations get older, they become more susceptible to age-related diseases such as Alzheimer’s Disease and Related Dementias (AD/ADRD), which affects more than 55 million individuals worldwide. The National Institutes of Health (NIH) aims to prevent and treat diseases such as AD/ADRD through generalist and disease-specific data resources that foster findable, interconnected, and interoperable data to accelerate biomedical innovations and medical interventions that improve human health. The goal of this assessment was to understand the value of AD/ADRD secondary data use from NIH genomic and multi-omics data resources: NIA Genetics of Alzheimer’s Disease Data Storage Site (NIAGADS), database of Genotypes and Phenotypes (dbGaP), AD Knowledge Portal (ADKP), and Gene Expression Omnibus (GEO). List of AD/ADRD datasets were retrieved through database searches or directly from repository staff (N=183). Citation frequency of data accessions were used to determine utilization of AD/ADRD genomic and multi-omics datasets in published literature. iSearch tool was used to assess secondary use drivers in resulting publications. Query of data accessions in Pubmed Central returned 284 unique publications. iSearch clustering by term showed that genomic datasets were used to study genetic risk factors of AD/ADRD while multi-omics datasets were used to functionally characterize genetic risk variants, identify gene regulatory networks, and biomarkers associated with neurodegenerative diseases. These findings indicate the usefulness of AD/ADRD secondary data to the scientific literature.

